# Spatiotemporal variability of dairy manure temperature during storage in earthen pits: Associations with meteorological factors

**DOI:** 10.1371/journal.pone.0347665

**Published:** 2026-05-07

**Authors:** Rana A. Genedy, Jactone A. Ogejo

**Affiliations:** Biological Systems Engineering Department, Virginia Tech, Blacksburg, Virginia, United States of America; North-Caucasus Federal University - Pyatigorsk Campus: Severo-Kavkazskij federal’nyj universitet Patigorskij institut filial, RUSSIAN FEDERATION

## Abstract

Manure storage is a critical component of nutrient management in dairy systems, but it is also a major source of greenhouse gas emissions and nutrient losses. Temperature regulates microbial activity and associated emissions; however, its spatial and temporal dynamics within stored manure are not well characterized. This study characterized the temporal and spatial variability of manure temperature in a dairy manure storage pit and examined its relationships with meteorological factors and management practices. Temperature was monitored at multiple vertical locations within the manure column over seasonal cycles, and statistical and machine-learning analyses were used to identify key drivers. Manure temperature exhibited clear seasonal patterns and vertical stratification, with differences of up to 10°C observed between lower and upper manure layers. During colder periods, higher temperatures were consistently observed in lower manure layers, whereas during warmer periods, temperatures near the surface sometimes approached or exceeded ambient air temperature. Temperature gradients are periodically reversed, producing turnover events that redistribute heat within the manure column, even in the presence of a surface crust. Ambient air temperature was the dominant external driver, explaining a substantial proportion of variability in manure temperature (R² = 0.72–0.84), while manure volume moderated the influence of atmospheric variability on internal temperatures. Other meteorological variables had comparatively smaller effects. These findings demonstrate that stored manure behaves as a vertically stratified thermal system governed by the interaction of atmospheric forcing and internal heat generation. Incorporating spatially resolved manure temperature dynamics into emission models can improve predictions of environmental impacts and inform more effective manure management strategies.

## Introduction

Increasing demand for dairy products, coupled with growing regulatory and societal expectations, is driving the adoption of more sustainable production practices in the dairy sector [1-4]. Within this context, manure management has emerged as a critical challenge. Dairy farms generate large quantities of manure, which, while rich in nutrients and valuable as a fertilizer, can also contribute to environmental degradation if not properly managed [5-7]. When effectively managed, manure can enhance soil fertility and support nutrient recycling within agricultural systems [8].

Manure storage is an essential component of on-farm nutrient management, enabling the strategic timing of land application. During storage, microbial decomposition produces volatile compounds, including ammonia, methane, and hydrogen sulfide. As a result, manure storage is a major source of greenhouse gases, particularly methane, accounting for approximately 5% to 30% of global agricultural methane emissions [9-11]. Ammonia volatilization can also result in substantial nitrogen losses, ranging from 5% to 50%, depending on management practices and environmental conditions [12,13]. Improving understanding of the physical and environmental conditions within stored manure is therefore critical for reducing nutrient losses and emissions and enhancing the retention of nutrient value.

Manure temperature is a key driver of physicochemical processes and microbial activity, directly influencing decomposition rates during storage [10]. Higher temperatures accelerate microbial metabolism, increasing emissions of greenhouse gases and other volatile compounds [10,11,14,15]. Field and modeling studies indicate that stored manure can reach temperatures significantly higher than ambient air, in some cases up to 10°C, due to internal heat generation and retention [16-19]. Despite this, many emission models rely on ambient air temperature as a proxy for manure temperature [20-24], introducing uncertainty in estimates of manure degradation and associated emissions. This limitation reflects a broader gap in field-based measurements of manure temperature dynamics and their coupling with meteorological drivers. The need for such data arises from the technical challenges of monitoring internal temperatures within storage systems, the spatial and temporal complexity of thermal processes within the manure mass, and the resulting reliance on simplified modeling assumptions.

Several environmental and management factors influence manure temperature during storage, yet their individual and combined effects remain poorly quantified. At the field scale, the daily addition of fresh manure introduces new biomass and heat, altering the existing temperature distribution within the storage system. Engineered covers and surface crusts act as insulating layers that modify heat exchange with the atmosphere [11,25–27]. Periodic agitation, typically performed prior to land application, redistributes heat and reduces temperature gradients, while partial pump-out practices alter manure volume and surface area, influencing the overall thermal profile [17]. Despite the recognized importance of these factors, there is a lack of comprehensive field-scale studies that quantify their individual contributions and interactions in governing manure temperature dynamics.

Consequently, this study characterizes the temporal and spatial patterns of manure temperature during storage and quantifies the relationships between these patterns and meteorological factors and management practices. These findings support improved emission modeling and inform management strategies to reduce nutrient losses and greenhouse gas emissions from manure storage.

## Materials and methods

### Study site

This study was conducted at a full-scale manure storage pit located on a dairy farm in Franklin County, Virginia, USA. Franklin County has a temperate climate with four distinct seasons and a mean elevation of ~365 m above sea level. The mean winter temperatures range from a high of 8.8 °C to a low of –3.4 °C (January), and mean summer temperatures range from a high of 30.3 °C to a low of 17.1 °C (July). The mean relative humidity ranges from 73% to 80% annually, with annual precipitation of approximately 1,120 mm. The growing season lasts roughly 180 days (April 20 to October 16).

The farm milks approximately 90 cows housed in a compost-bedded pack barn. Manure from the barn alleys is scraped twice daily and deposited into a non-perfect oval-shaped clay-lined earthen storage pit. The top surface area of the pit is about 990 m^2^, with dimensions of approximately 60 m by 27 m and an mean depth of 3.8 m. The storage can hold manure generated on the farm for approximately 120 days under typical conditions. Sometimes, manure is stored for extended periods if it cannot be applied because field conditions are deemed non-trafficable due to excessive soil moisture after rainfall, which poses a risk of soil compaction and equipment immobilization, or if the contractor who would apply the manure is unavailable. To avoid overflow when the pit nears capacity, the farm performs a partial pump-out to remove some manure and create additional storage volume until a complete pump-out can be done. In preparation for any pump-out, the manure is thoroughly agitated with a tractor-powered mixing pump to homogenize it. The manure is then transported via slurry trucks and applied to fields as fertilizer.

The study commenced in mid-February 2019, when the pit was approximately 30–40% full, and continued through seven consecutive storage periods (I to VII) over three years (2019–2022). Table 1 details each storage period along with the dates for partial and complete pump-out events. The seasons were defined as follows: winter, from December 21 to March 20; spring, from March 21 to June 20; summer, from June 21 to September 20; and fall, from September 21 to December 20. The periods were classified as cold (I, III, V, VII) and warm (II, IV, VI). Cold periods included late fall, winter, and early spring, specifically the months of December, January, February, March, and early April. During these months, the mean storage duration was 127 days (excluding Period I). In contrast, warm periods occurred in early spring, summer, and most of fall, spanning April through December. The mean storage duration during these warm months was 236 days.

**Table 1:** Manure storage periods, including start dates, partial pump-out dates, complete pump-out dates, and the seasons they cover.

Period and designation		Date			Seasons
		Start	Partial manure pump-out	Complete manure pump-out	
I	Cold	Feb 18, 2019	NA*	Apr. 08, 2019	Winter, Spring
II	Warm	Apr. 08, 2019	Aug. 23, 2019	Dec 26, 2019	Spring, Summer, Fall
III	Cold	Dec. 26, 2019	Mar. 04, 2020	Apr. 06, 2020	Winter, Spring
IV	Warm	Apr. 06, 2020	Jul. 07, 2020	Oct. 28, 2020	Spring, Summer, Fall
V	Cold	Oct. 28, 2020	N/A	Mar. 23, 2021	Fall, Winter
VI	Warm	Mar. 23, 2021	Oct. 18, 2021	Nov. 19, 2021	Spring, Summer, Fall
VII	Cold	Nov. 19, 2021	N/A	Mar. 31, 2022	Fall, Winter, Spring

*NA – not applicable.

### Instrumentation and parameter monitoring

The setup for monitoring manure temperature and weather parameters was described in Genedy and Ogejo [17]. Briefly, manure temperatures were measured using HOBO TMCx-HD sensors connected to HOBO UX120-006M 4-channel data loggers (Onset Computer Corp., Bourne, MA, USA). To measure manure temperatures in the storage pit, three rigid poles were installed along the pit’s major axis: near the manure inlet, at the midpoint, and near the outlet, where manure is pumped from storage. Each pole was graduated with depth markings to indicate the manure level (stage) within the pit and was equipped with four temperature sensors positioned at 45, 90, 168, and 244 cm above the pit bottom. These sensor positions are hereafter referred to as locations 045, 090, 168, and 244, respectively. Manure depth was defined as the distance from the pit bottom to the manure surface and varied over time as the stored volume changed. As the pit filled, sensors became progressively submerged in manure beginning with location 045 and continuing upward to location 244. Temperature data were considered valid only when the sensor at a given location was fully submerged in manure. For each location, the reported temperature represents the mean temperature across the three replicate sensors positioned at that location on the three poles. The locations were also considered to represent manure layers centered around them, with 045 representing the bottom layer and 244 representing the upper layer of the stored manure.

Meteorological parameters were monitored using an on-site weather station (DYACON® MS-130, DYACON Inc., Logan, UT, USA) installed on the bank of the manure storage pit. Sensors measuring ambient air temperature, wind speed, wind direction, relative humidity, and solar radiation were mounted approximately 3 m above ground level, while the rain gauge was installed at approximately 1 m above ground level. Sensor installation heights followed the manufacturer’s recommendations.

Manure temperature and meteorological data were recorded continuously at 30-min intervals throughout the study.

### Data handling and analysis

Prior to analysis, the data were screened for anomalous or inconsistent sensor readings. Raw manure temperature and meteorological data were examined for potential outliers associated with sensor malfunction. If the manure temperature at one location differed substantially from concurrent measurements recorded by other sensors at a similar location in the pit, it was flagged for review. Ambient air temperature readings inconsistent with expected regional conditions were similarly evaluated. Questionable measurements were cross-checked against records from the on-site weather station and the National Weather Service station at Roanoke Regional Airport (approximately 20 miles from the study site). Observations that could not be corroborated were removed. The remaining data were organized chronologically and verified to ensure correct alignment with the corresponding manure storage periods. The cleaned dataset was used for subsequent analyses.

Box plots were used to summarize the distributions of manure and ambient air temperatures across storage periods, showing medians, interquartile ranges, and variability to compare central tendency and dispersion. Differences in data quantity among locations reflected variation in sensor submergence duration; sensors positioned near the bottom of the pit remained submerged longer and therefore recorded more observations than sensors located in upper manure layers adjacent to the manure–ambient air interface.

### Statistical analysis

Analysis of variance was conducted to evaluate the effects of the storage Periods (I-VII) and location along the depth axis on manure temperature. Significant effects (p < 0.05) were followed by Tukey’s HSD test for pairwise comparisons. These statistical tests were performed using JMP Pro 18 (SAS Institute Inc., Cary, NC, USA).

The influence of meteorological factors on manure temperatures at different locations was evaluated using a combination of descriptive, inferential, and machine-learning approaches to capture linear, monotonic, and nonlinear relationships. To reduce temporal autocorrelation and heteroscedasticity, the 30-min measurements were aggregated into daily means prior to analysis. Wind direction was transformed into sine (east–west axis) and cosine (north–south axis) components to account for the circular nature of directional data. All correlation, regression, and machine-learning analyses were conducted separately for each measurement location.

Initial assessments of pairwise associations between manure temperatures at each measurement location and meteorological factors were conducted using Pearson correlation coefficients to quantify linear relationships and Spearman rank correlation coefficients to evaluate monotonic relationships. Pearson’s *r* was interpreted as: strong (∣r∣≥0.7), moderate (0.4≤∣r∣<0.7), and weak (∣r∣<0.4).

To quantify the independent contribution of meteorological factors while controlling for other predictors, standardized multiple regression models were estimated using ordinary least squares with z-scored predictors. Standardization (mean = 0, standard deviation = 1) enabled direct comparison of standardized regression coefficients (β) among predictors with different measurement units. Prior to regression modeling, variance inflation factors (VIFs) were calculated to assess multicollinearity among predictors, with values greater than 5 indicating potential multicollinearity. The relative importance of meteorological predictors was evaluated using variance decomposition based on the Lindeman–Merenda–Gold (LMG) method, which partitions the model R^2^ into contributions from each predictor while accounting for correlations among predictors [28].

Moderation analysis was conducted to test whether the volume of manure in storage influences the relationship between meteorological factors and manure temperature. The depth of manure in the storage pit was used as a proxy for manure volume. Hierarchical regression models were fitted in three steps. The base model included meteorological factors and manure depth:


$$T_{m,h}=\beta_{\text{0}}+\sum \beta_{i}W_{i}+\beta_{j}d_{m}+\varepsilon$$

where *T*_*m,h*_ is manure temperature at location *h* (045, 090, 168, 244), *W*_*i*_ represents meteorological predictors (ambient air temperature, wind speed, wind direction components, solar radiation, and rainfall), *d*_*m*_ is manure depth used as a proxy for manure volume, *β*_*0*_ is the intercept, *β*_*i*_ and *β*_*d*_ are regression coefficients and ε is the residual error.

Two-way interaction terms between manure depth and meteorological factors were then added:


$$T_{m,h}=\beta_{\text{0}}+\sum \beta_{i}W_{i}+\beta_{j}d_{m}+\sum \beta_{k}\left(W_{i}\times d_{m}\right)+\varepsilon$$

where *β*_*id*_ represents the regression coefficients for the two-way interaction terms between each meteorological factor and manure depth.

Finally, three-way interaction terms among meteorological factors and manure depth were incorporated:


$$T_{m,h}=\beta_{\text{0}}+\sum \beta_{i}W_{i}+\beta_{j}d_{m}+\sum \beta_{k}\left(W_{i}\times d_{m}\right)+\sum \beta_{i,j}\left(W_{i}\times W_{j}\times d_{m}\right)+\varepsilon$$

where *β*_*ijd*_ represents the regression coefficients for the three-way interaction terms involving pairs of meteorological factors and manure depth.

The contribution of predictors added at each step was evaluated using the change in explained variance ($\Delta R^{\text{2}}$) and associated F-tests.

To explore potential nonlinear and interaction-driven relationships, a Gradient Boosting Regressor was implemented [29]. Predictor importance within the Gradient Boosting Regressor model was evaluated using permutation-based feature importance, which quantifies the reduction in predictive performance ($R^{\text{2}}$) when the values of a predictor are randomly permuted.

All statistical and machine-learning analyses were performed in Python (version 3.12). Hypothesis testing, data transformations, and correlation analyses were conducted using the *SciPy* library [30]. Standardized regression models, variance inflation factor calculations, moderation interaction models, and F-tests were implemented using the *statsmodels* library [31]. Machine-learning models, including the Gradient Boosting Regressor, data scaling procedures, and permutation-based feature importance, were implemented using *scikit-learn* [32].

## Results

### Daily manure and ambient air temperatures patterns

**Fig 1:**
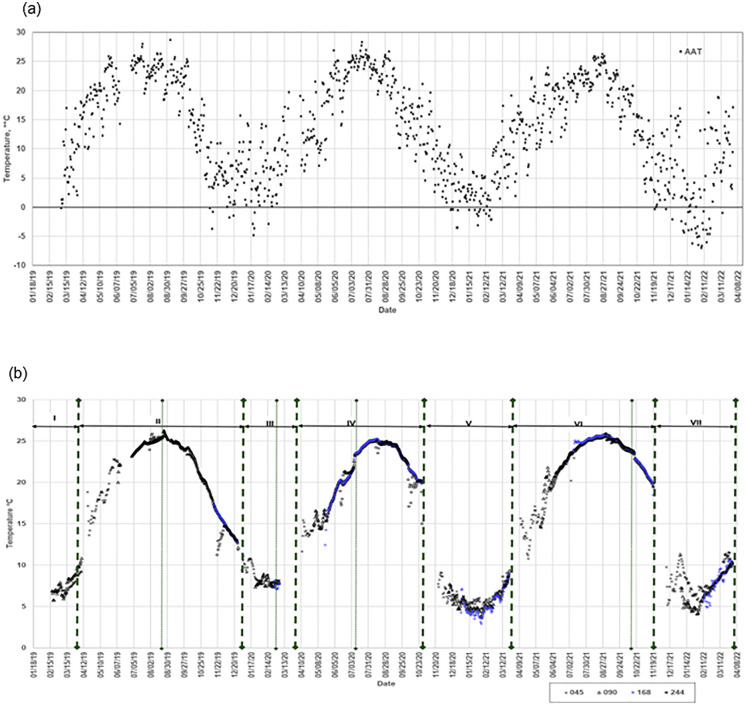
(a)The daily mean ambient air temperature, AAT; (b) manure temperatures at the different locations along the depth axis. The vertical dashed lines represent when total pump-outs occurred. The dotted lines indicate partial pump-out events. The storage periods are labeled I – VII.

The daily mean ambient air temperature and manure temperatures at the different locations during the study are shown in Fig 1. Period I was the shortest, lasting only 49 days, due to the study starting on February 18, 2019, which coincided with the mid-storage cycle when the manure pit was at about 30–40% capacity (S1 Fig). Thus, temperature readings for the locations 045 and 090 were available at the start of the study. Each storage period concluded with a manure pump-out event, shown by the vertical dashed lines in Fig 1b. The pump-out events also marked the start of the next period, which was characterized by low manure volumes in the pit (S2 Fig).

Temperature trends during storage periods exhibited clear diurnal patterns that distinguished cold from warm periods. During cold periods, manure temperatures were consistently lower across all locations, reflecting corresponding ambient air temperatures. The bottom layer (045) exhibited the highest temperatures, whereas the top layer (244) showed greater variability and more rapid temperature changes, closely tracking fluctuations in ambient air temperature (Fig 1b). In contrast, during warm periods, a different stratification pattern was observed. Temperatures in the bottom  layers were consistently lower than those in the upper layers. During warm periods, manure temperatures increased steadily across all locations, with the top layer (244) reaching or exceeding ambient air temperatures during mid- to late summer (August–September).

Manure temperatures consistently lagged changes in ambient air temperature and exhibited more gradual fluctuations. Partial and total pump-out events (e.g., S3 and S4 Figs) temporarily disrupted temperature stratification by altering the existing temperature gradient. In contrast, full pump-out events eliminated the temperature gradient during complete emptying of the pit. Following emptying, the gradient was reset, consistent with manure accumulation during the subsequent storage period.

### Thermal turnover

**Fig 2:**
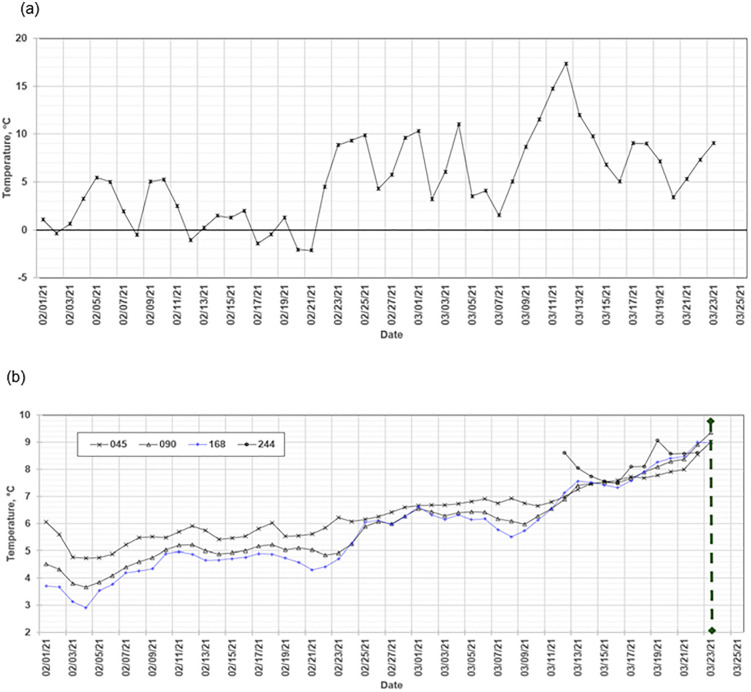
(a) Daily mean ambient air temperature, AAT; and (b) daily mean manure temperature at various locations along the depth axis showing a thermal turnover event during a cold storage period.

**Fig 3:**
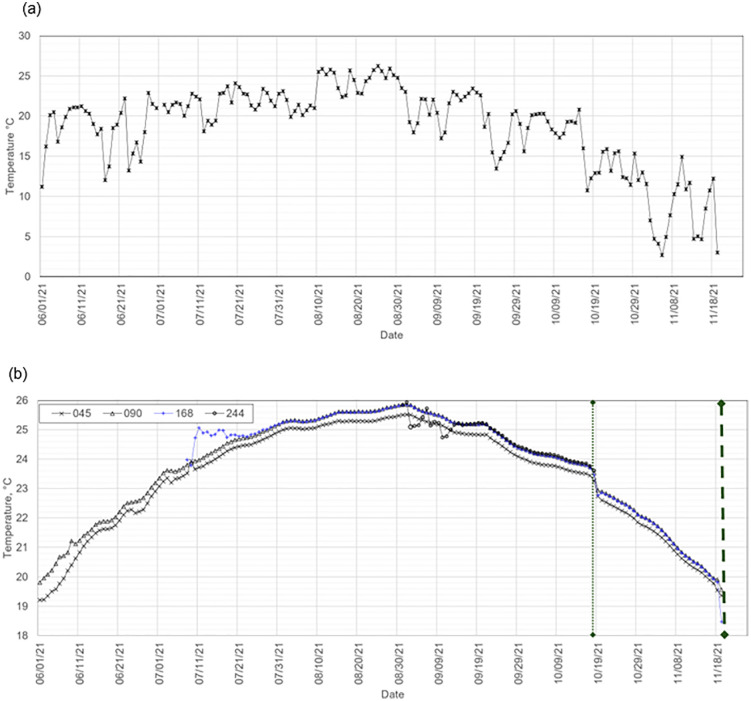
(a) Daily mean ambient air temperature, AAT; (b) daily mean manure temperature at various locations along the depth axis showing a thermal turnover event during a warm storage period.

The mean manure temperatures at various depth locations revealed clear seasonal thermal dynamics within the storage pit. During the cold periods, particularly in the first two weeks of March, a thermal turnover occurred in the bulk volume of the stored manure slurry (e.g., Fig 2b). This turnover was characterized by the temperatures of the 045-, 090-, and 168-locations converging to a common value. Following this, the thermal gradient of the layers reversed, with the 045 location, which was initially the warmest, becoming the coldest. Interestingly, this thermal turnover occurred despite the presence of a crust on the stored liquid (S4–S8 Figs). During this period of thermal turnover, the daily mean ambient air temperature ranged from 1 °C to 17 °C. These temperature fluctuations are typical for the region as the seasons transition from winter to spring. In contrast, during warm periods, particularly in early November, a similar convergence of temperatures was observed (Fig 3b), consistent with conditions preceding turnover; however, a complete turnover was not observed, and partial pump-out events involving mechanical mixing were conducted during this period (S3 Fig).

### Manure temperature by storage period

**Fig 4:**
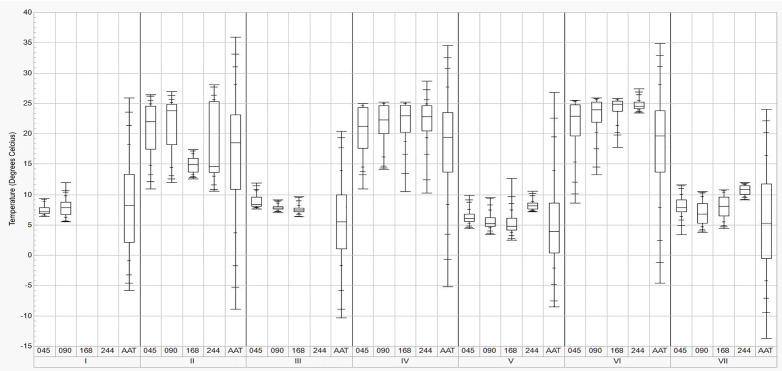
Distribution of manure temperatures at various depth locations (045 = 0.45 m, 090 = 0.90 m, 168 = 1.68 m, and 244 = 2.44 m above the pit bottom) and ambient air temperature (AAT) across storage periods I–VII.Boxes represent the interquartile range (25th–75th percentile), with the median shown by the horizontal line.

Manure and ambient air temperatures during the study period are shown in Fig 4, with summary statistics and statistical comparisons provided in Table 2. Temperature patterns differed clearly between cold and warm storage periods, reflecting seasonal meteorological conditions and the thermal behavior of stored manure.

**Table 2:** Mean manure temperatures at various depth locations and ambient air temperatures (± SE) during each storage period (I–VII). Temperatures within each column with the same letter are not significant. The average temperatures within each row (Period) with the same symbol are not significantly different (Tukey HSD, p < 0.05).

Period	Manure temperature, °C				Ambient air temperature, °C
	045	090	168	244	
I	7.44 ± 0.08^E,#^ (2,343)	7.91 ± 0.08^C,%^ (2,339)	- NA -	- NA -	8.35 ± 0.09^C,*^ (1,586)
II	20.74 ± 0.06^B,#^ (11,047)	21.50 ± 0.07^B,%^ (8,603)	14.90 ± 0.14^A,$^ (1,878)	18.47 ± 0.12^A,&^ (2,656)	16.84 ± 0.06^B,*^ (11,520)
III	8.87 ± 0.07^C,#^ (3,043)	7.87 ± 0.09^C,%^ (1,897)	7.53 ± 0.18^B,%^ (434)	- NA -	5.72 ± 0.07^D,*^ (3,295)
IV	20.65 ± 0.05^B,#^ (9,585)	21.59 ± 0.05 ^B,%^ (8,668)	22.29 ± 0.05^C,$^ (7,739)	22.30 ± 0.07^B,$^ (4,774)	18.47 ± 0.04 ^A,*^ (10,506)
V	6.27 ± 0.05^F,#^ (5,551)	5.62 ± 0.05^E,%^ (4,884)	5.26 ± 0.06^D,$^ (3,907)	8.19 ± 0.16^C,&^ (488)	4.92 ± 0.04^E,*^ (6,283)
VI	21.56 ± 0.05^A,#^ (10,657)	23.30 ± 0.05^A,%^ (9,007)	24.16 ± 0.06^E,$^ (6,452)	24.69 ± 0.10^D,&^ (2,297)	18.57 ± 04 ^A,*^ (11,569)
VII	8.07 ± 0.02^D,#^ (5,283)	6.97 ± 0.03 ^D,$^ (4,206)	8.03 ± 0.04 ^F,#^ (2,419)	10.76 ± 0.10^E,&^ (738)	5.69 ± 0.10 ^D,*^ (6,350)

NA indicates no data (manure temperature sensor not submerged). Numbers in parentheses represent the number of observations.

### Cold periods

During the cold storage periods (I, III, V, and VII), manure temperatures were lower and less variable. Overall, mean manure temperatures differed significantly among storage periods and locations (p < 0.001; Table 2). Across all measurement locations, the lowest temperatures consistently occurred during Period V. Notably, during Periods I and III, higher temperatures were observed at locations 090 and 045, respectively, whereas during Period VII, locations 168 and 244 exhibited higher temperatures. Within individual cold storage periods, temperatures varied along the depth axis. Specifically, in Period I, the highest temperatures occurred at location 090 and the lowest at 045, whereas in Period III, location 045 exhibited the highest temperatures and location 168 the lowest. Similarly, during Periods V and VII, the upper location (244) generally exhibited higher temperatures relative to mid‑depth locations.

### Warm Periods

During the warm storage periods (II, IV, and VI), manure temperatures exhibited greater variability than during cold periods, particularly at the upper location (244). Overall, mean manure temperatures differed significantly among locations during the warm periods (p < 0.001; Table 2). Across locations, variability at the upper location (244) was most pronounced during Periods II and IV and was reduced during Period VI. Notably, during Period II, temperatures varied along the depth axis, with mid‑depth locations exhibiting lower temperatures relative to other locations. During Period IV, higher temperatures were observed at the upper location, while lower locations remained cooler. By comparison, during Period VI, temperatures were elevated across all locations, with a pronounced vertical gradient near the manure surface. Additionally, partial pump‑out events during Periods IV and VI coincided with changes in temperature distributions at intermediate and upper locations.

### Influence of meteorological factors and pit volume on manure temperature

**Fig 5:**
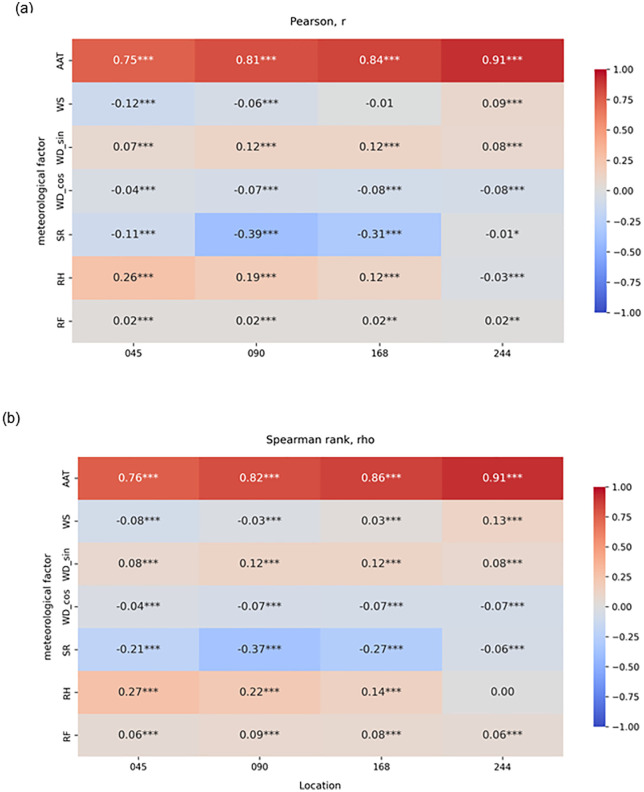
Correlation heatmaps showing relationships between meteorological factors and manure temperature at locations 045 (0.45m), 090 (0.90 m), 168 (1.68 m), and 244 (2.44 m) above the bottom of the storage pit. Color gradients indicate the strength and direction of correlations, with red denoting positive and blue negative. Panel (a) shows Pearson correlation coefficients (linear relationships), and panel (b) shows Spearman rank coefficients (monotonic relationships). Statistical significance is indicated by asterisks (* p < 0.05, ** p < 0.01, *** p < 0.001). Meteorological variables include ambient air temperature (AAT), wind speed (WS), wind direction components (WD_sin and WD_cos), solar radiation (SR), relative humidity (RH), and rainfall (RF).

Pearson and Spearman correlations yielded nearly identical patterns between manure temperature and the examined meteorological factors (Figs 5a and 5b), indicating largely monotonic and approximately linear relationships. Across all locations at various depths, ambient air temperature showed the strongest positive association with manure temperature. Solar radiation exhibited moderate negative correlations at several locations, whereas relative humidity showed weak positive associations. Wind speed, wind direction components, and rainfall exhibited minimal relationships with manure temperature. Although several correlations were statistically significant, the magnitude of the coefficients indicates that ambient air temperature was the only meteorological factor strongly associated with manure temperature.

Variance inflation factors ranged from 1.03 to 1.63 across the four locations, indicating minimal multicollinearity among predictors. Standardized regression analysis (Table 3) identified ambient air temperature as the dominant predictor of manure temperature across all locations, with strong positive effects that increased toward location 244. Relative humidity and manure volume were also significant positive predictors, although their influence declined toward location 244. Solar radiation exhibited a consistent negative association across all locations, with the strongest effect observed at location 090. Wind speed showed weak negative effects at locations 045–168 but was not significant at location 244, while wind direction components and rainfall had negligible and inconsistent effects. Overall, predictor influence varied by location, and meteorological drivers, particularly ambient air temperature, exerted the greatest control at upper locations.

**Table 3:** Standardized regression coefficients for meteorological factors of manure temperature across measurement locations along the depth axis.

Predictor	Location											
	045			090			168			244		
	beta	SE	p-value	beta	SE	p-value	beta	SE	p-value	beta	SE	p-value
AAT	0.7435	0.0026	p < 0.001	0.7096	0.0028	p < 0.001	0.7754	0.0028	p < 0.001	0.8954	0.0020	p < 0.001
WS	−0.0541	0.0031	p < 0.001	−0.0260	0.0032	p < 0.001	−0.0146	0.0032	p < 0.001	0.0013	0.0024	p = 0.599
WD_sin	−0.0365	0.0026	p < 0.001	−0.0215	0.0027	p < 0.001	−0.0188	0.0028	p < 0.001	0.0049	0.0020	p = 0.012
WD_cos	0.0242	0.0026	p < 0.001	0.0012	0.0026	p = 0.639	0.0037	0.0027	p = 0.168	0.0181	0.0019	p < 0.001
SR	−0.0691	0.0026	p < 0.001	−0.1826	0.0028	p < 0.001	−0.0853	0.0029	p < 0.001	−0.0387	0.0020	p < 0.001
RH	0.2585	0.0032	p < 0.001	0.1350	0.0032	p < 0.001	0.0943	0.0033	p < 0.001	0.0051	0.0024	p = 0.035
RF	−0.0136	0.0026	p < 0.001	−0.0104	0.0026	p < 0.001	−0.0086	0.0026	p < 0.001	−0.0056	0.0020	p = 0.005
dm	0.2285	0.0026	p < 0.001	0.2592	0.0027	p < 0.001	0.2317	0.0027	p < 0.001	0.1251	0.0020	p < 0.001

AAT- ambient air temperature, WS – wind speed, WD_sin and WD_cos – wind direction components, SR – solar radiation, RH – relative humidity, RF – rainfall, dm – manure depth (proxy for volume of manure in storage). beta - standardized coefficient and SE - standard error.

Variance decomposition (Table 4) showed that ambient air temperature dominated the explained variance in manure temperature across all locations, accounting for 66–94% of the total R^2^, with the strongest contribution at location 244. Manure volume was the second most influential predictor (5–16%), followed by smaller contributions from relative humidity and solar radiation, which were more pronounced at lower and intermediate locations but diminished toward location 244. Wind speed, wind direction components, and rainfall each contributed negligibly (<2%) across all locations. Collectively, these results confirm the dominant role of ambient air temperature and manure volume in explaining variability in manure temperature.

**Table 4:** Variance decomposition of meteorological predictors explaining manure temperature across four locations.

Predictor	Location Variance (%)			
	045	090	168	244
AAT	74.79	66.13	76.16	93.76
WS	1.91	0.68	0.35	0.48
WD_sin	0.30	0.71	0.72	0.42
WD_cos	0.21	0.32	0.39	0.37
SR	1.29	12.48	6.86	0.14
RH	8.76	3.34	1.38	0.08
RF	0.05	0.04	0.03	0.02
dm	12.68	16.28	14.11	4.73

AAT- ambient air temperature, WS – wind speed, WD_sin and WD_cos – wind direction components, SR – solar radiation, RH – relative humidity, RF – rainfall, dm – manure depth (proxy for volume of manure in storage).

Moderation analysis (Table 5) showed that interaction terms between meteorological predictors and manure volume improved explanatory power across all locations. Base models explained 73–84% of the variance in manure temperature, increasing to 74–89% with two‑way interactions and to 78–90% with three‑way interactions. The greatest improvement occurred at location 168 (ΔR² ≈ 0.10), while gains at other locations were smaller, particularly at location 244 (ΔR² ≈ 0.05).

**Table 5:** Model fit statistics for models evaluating the moderating effect of manure volume on the relationship between meteorological predictors and manure temperature across four locations.

Location	Models R^2^		
	Base	Two-Way	Three-Way
045	0.7255	0.7375	0.7805
090	0.7994	0.8427	0.8553
168	0.7907	0.8867	0.8962
244	0.8448	0.8894	0.8947

Manure temperature at locations 045 (0.45 m), 090 (0.90 m), 168 (1.68 m), and 244 (2.44 m) above the bottom of the storage pit.

**Fig 6:**
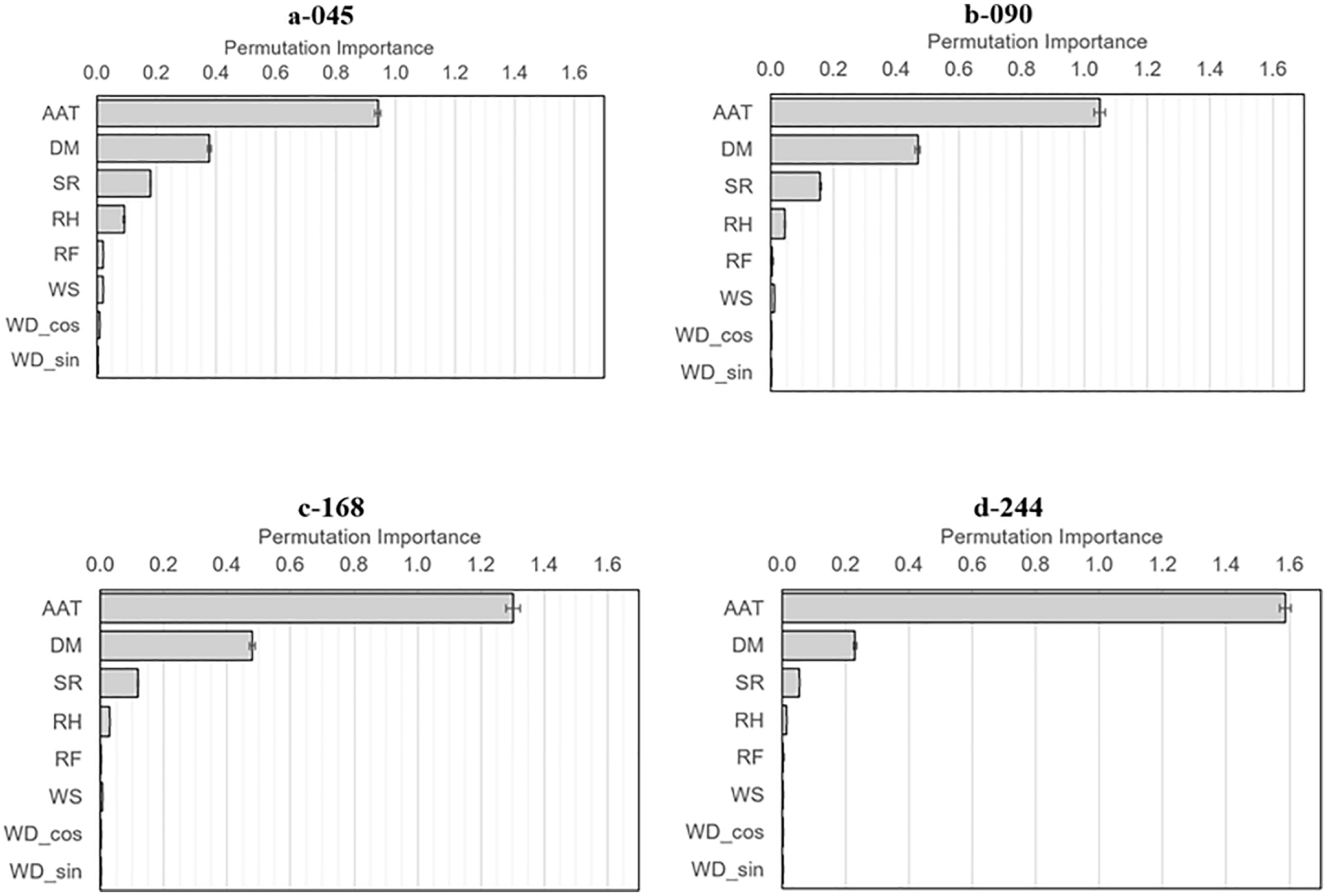
Permutation importance from Gradient Boosting Regressor models predicting manure temperature at locations 045, 090, 168, and 244 along the depth axis. Meteorological factors predictors include ambient air temperature (AAT), wind speed (WS), wind direction (WD_sin and WD_cos), solar radiation (SR), relative humidity (RH), rainfall (RF), and manure depth (dm; proxy for manure volume).

Gradient Boosting Regressor models further improved predictive performance relative to linear regression across all locations (Table 6), increasing R² from 0.72–0.84 to 0.89–0.96. The largest gains were observed at locations 045, 168, and 090, whereas improvements at location 244 were smaller. Permutation importance analysis (Fig 6) revealed consistent predictor rankings across all locations, with air temperature as the dominant predictor, followed by manure volume. Solar radiation and relative humidity contributed modestly, particularly at lower and intermediate locations, while wind-related variables and rainfall had minimal influence. These patterns were consistent with regression and variance decomposition results, reinforcing the central role of ambient air temperature and manure volume.

**Table 6:** Performance comparison (R^2^) between standard Linear Regression and non-linear Gradient Boosting Regressor of manure temperature across locations.

Location	Linear R²	Gradient Boosting R²
045	0.7235	0.9208
090	0.8012	0.9585
168	0.7904	0.9616
244	0.8445	0.8898

Manure temperature at locations 045 (0.45 m), 090 (0.90 m), 168 (1.68 m), and 244 (2.44 m) above the bottom of the storage pit.

## Discussion

This study examined temperature dynamics of dairy manure during storage in a clay pit and identified clear seasonal patterns, including thermal stratification and periodic turnover. Stratification varied between the cold and warm periods and was influenced by meteorological conditions and management practices. During cold periods, higher temperatures were consistently observed in manure layers at the bottom of the storage pit. During warm periods, near-surface manure layers approached or exceeded ambient air temperature, perhaps due to direct solar heating. Elevated temperatures at the bottom layers likely reflect reduced heat loss, limited mixing, and heat generation from microbial activity within the manure mass.

Although thermal stratification in manure storage systems has received limited direct study, related research provides indirect evidence of similar processes. Studies of greenhouse gas and ammonia emissions highlight the importance of environmental controls on manure systems [25,33-35]. For example, ammonia emissions from dairy slurry increase with ambient temperature and wind speed and decrease with precipitation [25,36,37]. Although manure temperature was not measured in those studies, the strong association observed here between manure and ambient air temperatures supports the role of meteorological forcing as a primary control in regulating manure processes. Additional indicators of stratification include nutrient and gas turnover during storage and methane release during winter thaw events [33–35,38,39].

Turnover events observed in this study are consistent with density-driven mixing processes described in stratified aquatic systems. Temperature gradients can destabilize the manure column and induce vertical mixing, which redistributes heat and dissolved constituents [40–43]. A similar mechanism likely operates in manure storage systems. Turnover occurred even in the presence of a surface crust, indicating that mixing can persist beneath surface barriers. Crust formation modifies heat transfer and reduces the influence of solar radiation and wind at the surface [13,44,45]. However, it did not prevent internal mixing, and it likely reduced the intensity of turnover. These mixing events can transport dissolved gases, such as methane and ammonia, from deeper layers to the surface, leading to episodic releases [33,35]. Management practices also influenced thermal behavior. Partial pump-out events during Warm periods disrupted stratification and prevented complete turnover, showing that operational interventions can alter both thermal structure and emission dynamics.

Variance decomposition showed that meteorological variables explained manure temperature variation, with ambient air temperature contributing 66–94% of the total model R² across locations. This reflects efficient thermal coupling between the manure surface and the atmosphere, whereby temperature gradients drive continuous heat exchange across the air–slurry interface, causing manure temperatures to closely follow temporal variations in ambient conditions through convective and radiative fluxes, with evaporation further modulating surface energy balance. Regression and variance decomposition analyses reinforced this relationship and identified manure volume as a key moderating factor, reflecting the influence of thermal inertia and reduced responsiveness to short-term atmospheric forcing in larger storage systems. Wind speed was negatively associated with manure temperature, consistent with enhanced convective heat loss, while relative humidity showed positive associations, reflecting reduced evaporative cooling and greater heat retention. These relationships are consistent with the surface energy balance processes that govern heat and mass transfer in manure systems [18].

In contrast to the dominant influence of air temperature, solar radiation exhibited a moderate inverse association with manure temperature, suggesting that under certain conditions, enhanced evaporation and surface cooling may offset radiative heat inputs and limit heat penetration into the slurry. These surface-driven processes can induce thermal stratification and constrain microbial heat generation within the bulk, although biological contributions remain secondary and condition-dependent [46-49]. Compared with air temperature and solar radiation, other meteorological variables, including relative humidity, wind speed, wind direction, and rainfall, showed weaker relationships with manure temperature, consistent with their primary role in modulating surface heat exchange rather than internal heat generation [18,19,49].

The volume of manure in storage further moderated the influence of meteorological drivers by introducing depth-dependent thermal responses within the slurry. Near-surface layers exhibited stronger and more immediate responses to atmospheric conditions, whereas bottom layers were less sensitive to short-term variability, indicating that temperature fluctuations attenuate with depth due to limited mixing and vertical heat transfer. Similar vertical patterns have been reported in manure storage systems, where surface layers respond rapidly to environmental forcing while deeper layers remain more thermally stable due to thermal buffering and conductive heat transfer [18,50], with comparable gradients observed in other organic waste systems [51]. These results indicate that stored manure functions as a thermal buffer, with larger volumes increasing thermal inertia and dampening short-term atmospheric fluctuations. The improved performance of the Gradient Boosting Regressor relative to linear models further suggests that manure temperature dynamics reflect nonlinear interactions among environmental drivers, particularly in the bottom layers where responses are delayed and influenced by cumulative thermal processes [17]. Permutation importance analysis confirmed ambient air temperature as the dominant predictor across all locations, followed by manure volume, with solar radiation exerting a secondary influence and other variables contributing comparatively minor effects, consistent with mechanistic studies showing that manure temperature distributions are primarily controlled by surface energy exchange and vertical heat transfer [18].

Although this study provides detailed insight into manure temperature dynamics, several limitations should be considered. Direct measurements of gas emissions were not collected, limiting the ability to assess how thermal stratification and turnover may influence emission processes. In addition, observations were derived from a single storage system, and variability in manure composition, climatic conditions, storage design, and management practices may affect the broader applicability of these findings. Finally, while statistical and machine-learning approaches identify strong associations among variables, they do not fully resolve causal relationships or explicitly represent the underlying physical and biological processes that govern temperature dynamics.

Overall, manure storage systems can be conceptualized as vertically stratified thermal systems in which temperature dynamics reflect the combined influence of atmospheric forcing and manure volume. Ambient air temperature drives temporal variability, while manure volume governs the extent to which these external signals propagate through the manure column. These interactions can lead to seasonal stratification and periodic redistribution of heat within the system. Because manure temperature regulates microbial activity, nutrient transformations, and gaseous emissions, improved understanding of these dynamics can support the development of more accurate predictive models and inform management strategies to reduce environmental impacts.

## Conclusion

This study characterized temperature dynamics in a manure storage pit and demonstrates that manure storage systems can be conceptualized as vertically stratified thermal systems with distinct seasonal patterns. During cold periods, higher temperatures were consistently observed in the manure layers at the bottom of the storage pit, whereas during warm periods, near‑surface layers sometimes approached or exceeded ambient air temperature, consistent with episodic surface warming. Temperature gradients were occasionally reversed, producing turnover events that redistributed heat within the manure mass, even in the presence of a surface crust.

Statistical and machine‑learning analyses identified ambient air temperature as the primary environmental driver of manure temperature, while manure volume moderated the propagation of meteorological variability through the manure column. Together, these results indicate that meteorological conditions and storage characteristics jointly control thermal behavior in manure storage systems. Because manure temperature regulates microbial activity, nutrient transformations, and gaseous emissions, incorporating empirically derived thermal dynamics, particularly air–manure temperature coupling and volume effects, into manure management and emission modeling frameworks can improve predictions of environmental impacts. This integrated understanding of manure thermal behavior provides a foundation for developing more realistic manure management and emission modeling frameworks, improving the ability to quantify thermal conditions and associated nutrient and gaseous losses during storage.

## Supporting information

S1 Fig Manure storage pit at the initiation of study, February 18, 2019, during the installation of the manure temperature sensors with manure level at 30–40% capacity. (TIFF)

S2 Fig Example manure level in pit one week after a total pump-out. (TIFF)

S3 Fig Video of the mixing process before removing manure for land application. This process was conducted for the partial and total pump-out events.
(MOV)

S4 Fig Manure in pit after mixing is complete and ready for pump out for land application. (TIFF)

S5 Fig Example storage pit manure contents when 50% full during a cold period. (TIFF)

S6 Fig Example storage pit manure contents when 50% full during a warm period. (TIFF)

S7 Fig Example storage pit manure contents when completely full during a cold period. (TIFF)

S8 Fig Example storage pit manure contents when completely full during a warm period. (TIFF)
