# Supplementary figures and images for: Spatiotemporal variability of dairy manure temperature during storage in earthen pits: Associations with meteorological factors
Source: PLoS One. 2026 May 7;21(5):e0347665. doi: 10.1371/journal.pone.0347665 (PMC13152168; doi:10.1371/journal.pone.0347665)

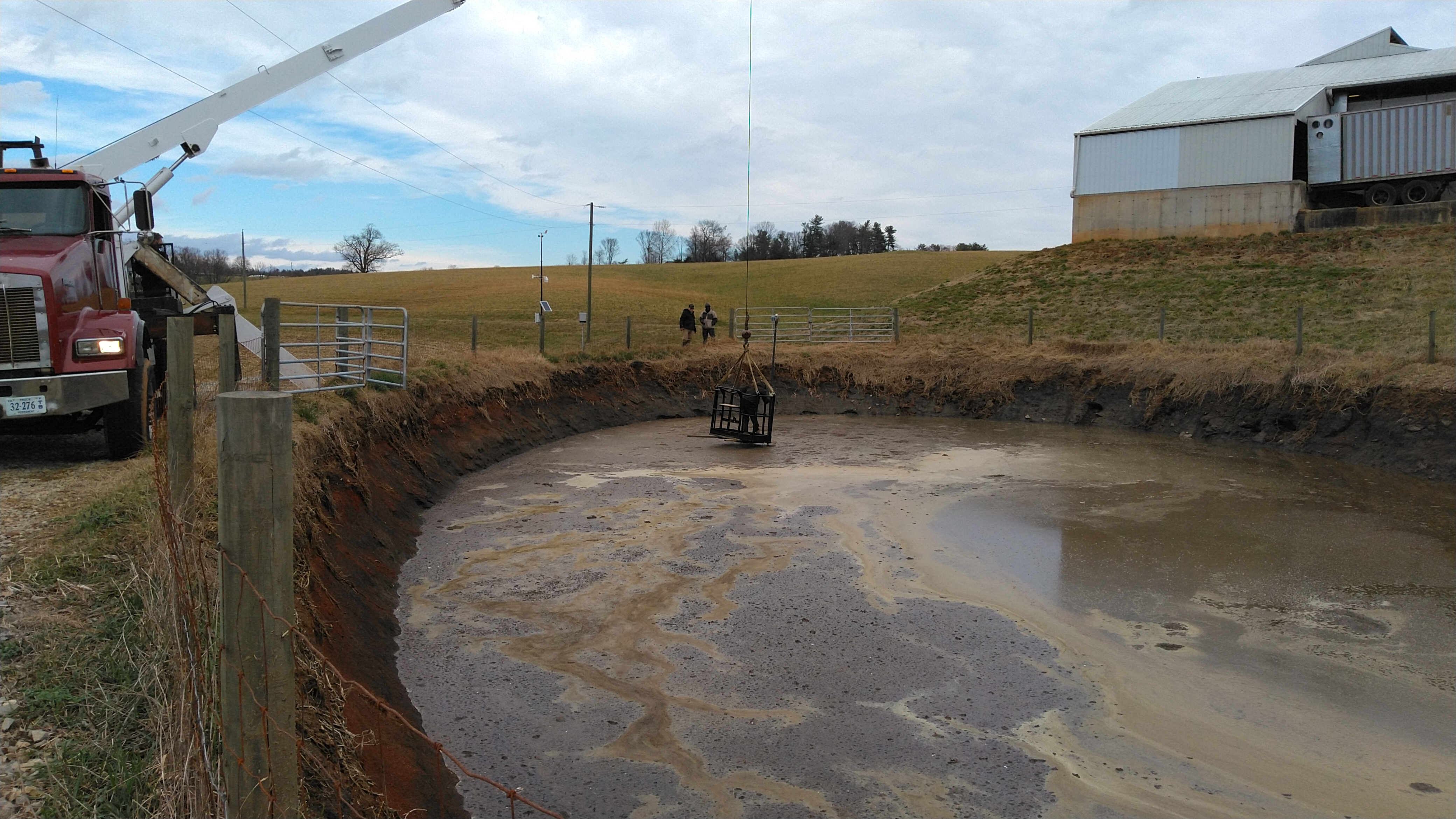

Supplement: S1 Fig — (TIFF) [file pone.0347665.s001.tiff]

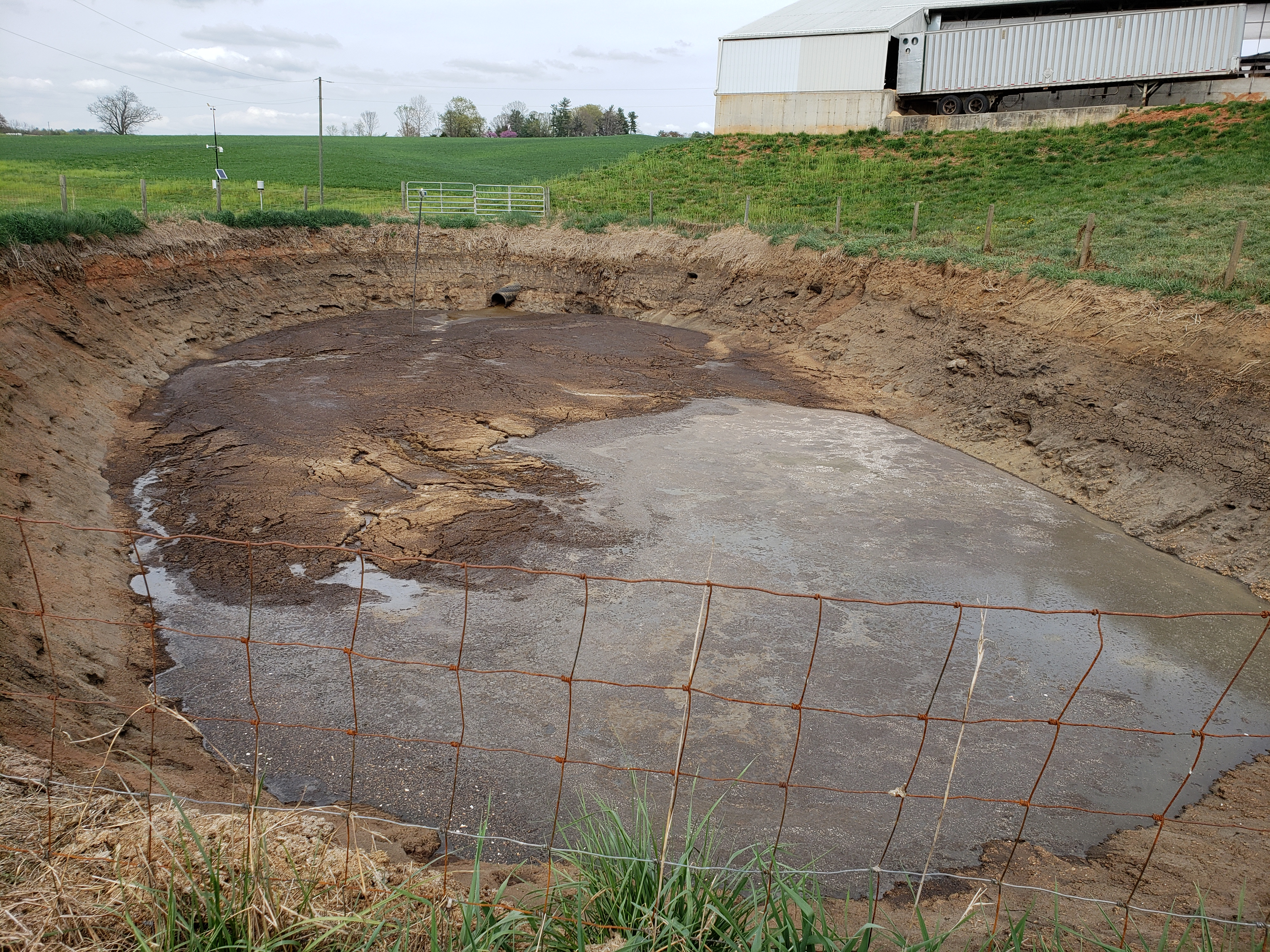

Supplement: S2 Fig — (TIFF) [file pone.0347665.s002.tiff]

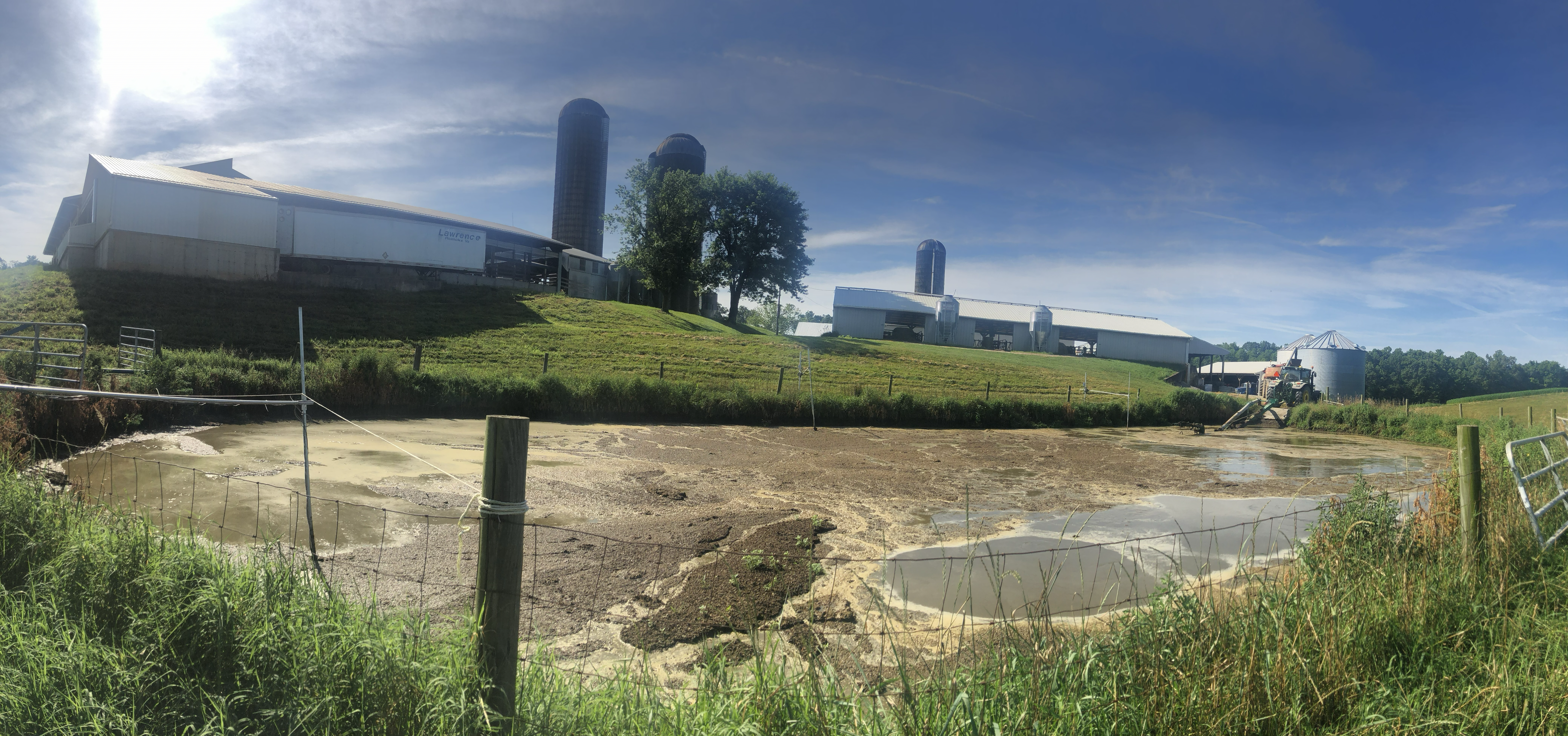

Supplement: S4 Fig — (TIFF) [file pone.0347665.s004.tiff]

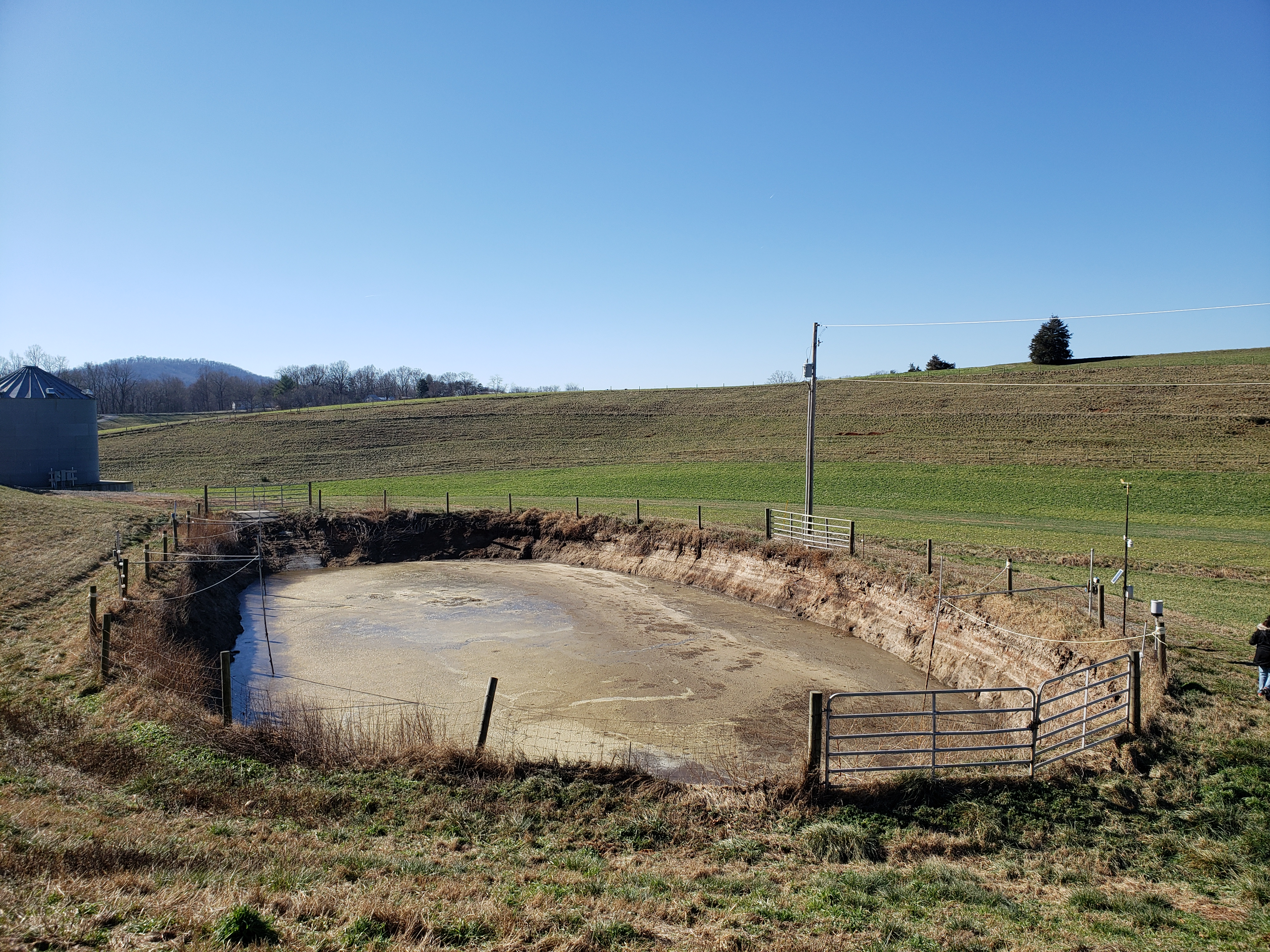

Supplement: S5 Fig — (TIFF) [file pone.0347665.s005.tiff]

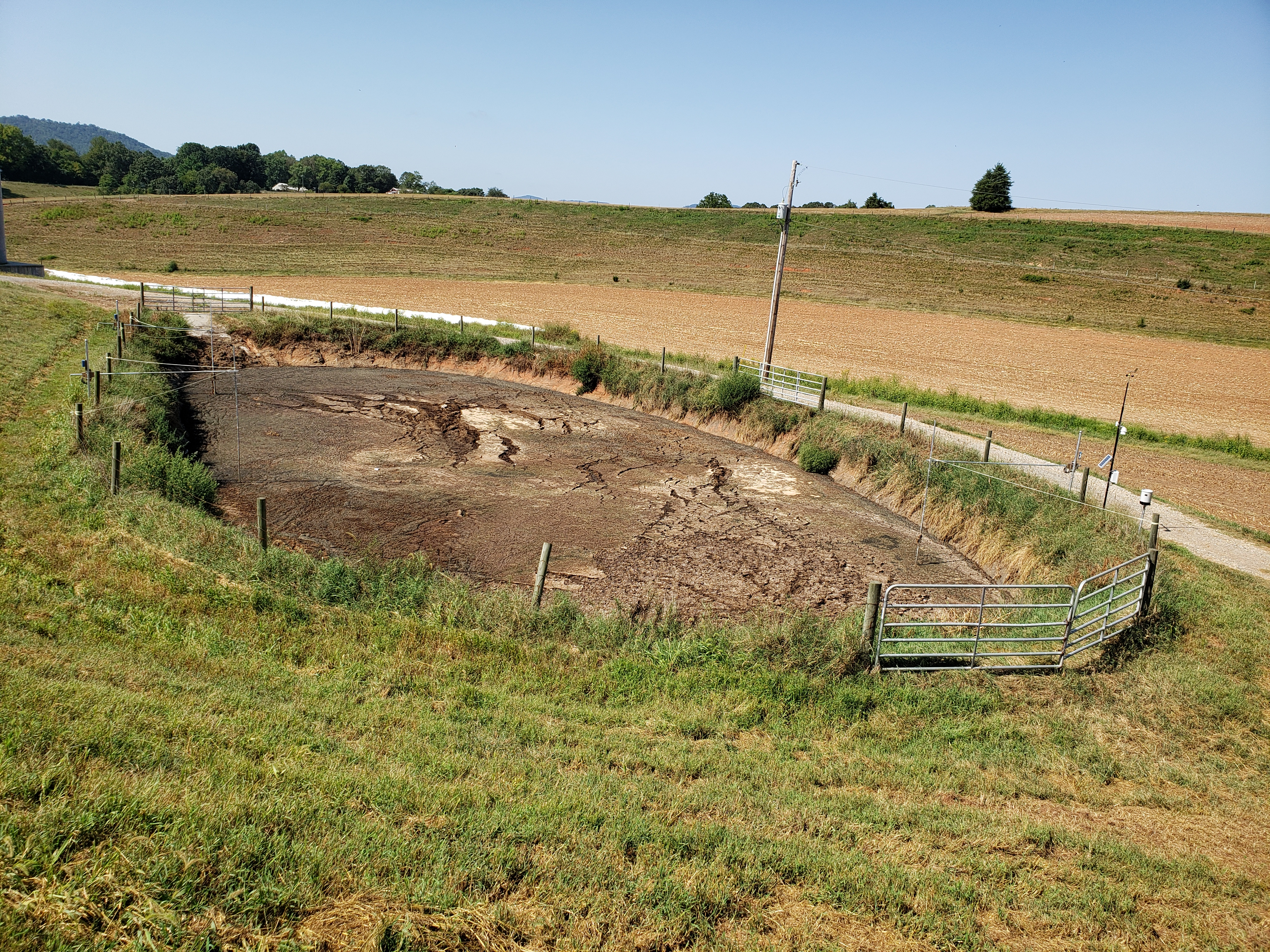

Supplement: S6 Fig — (TIFF) [file pone.0347665.s006.tiff]

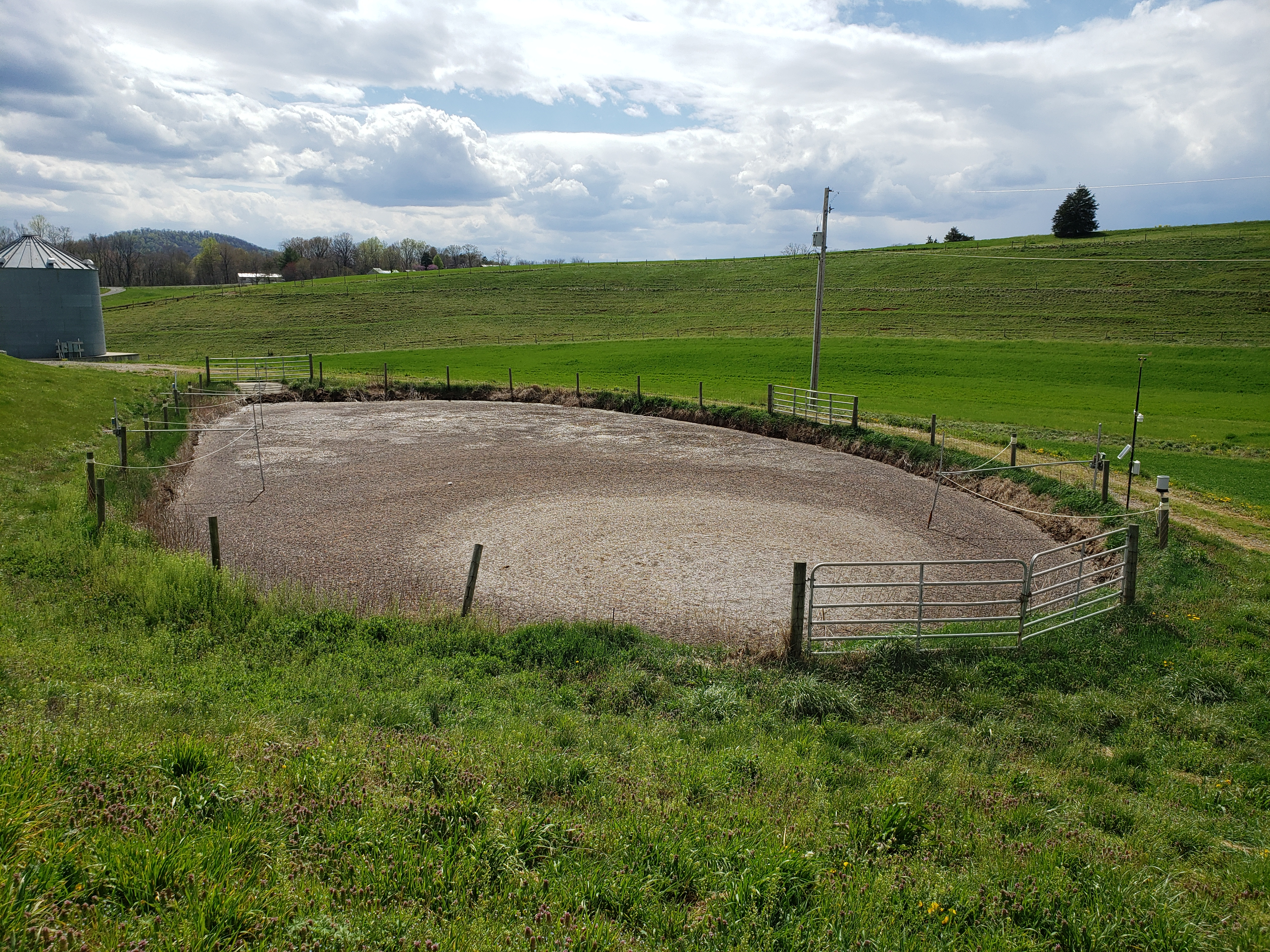

Supplement: S7 Fig — (TIFF) [file pone.0347665.s007.tiff]

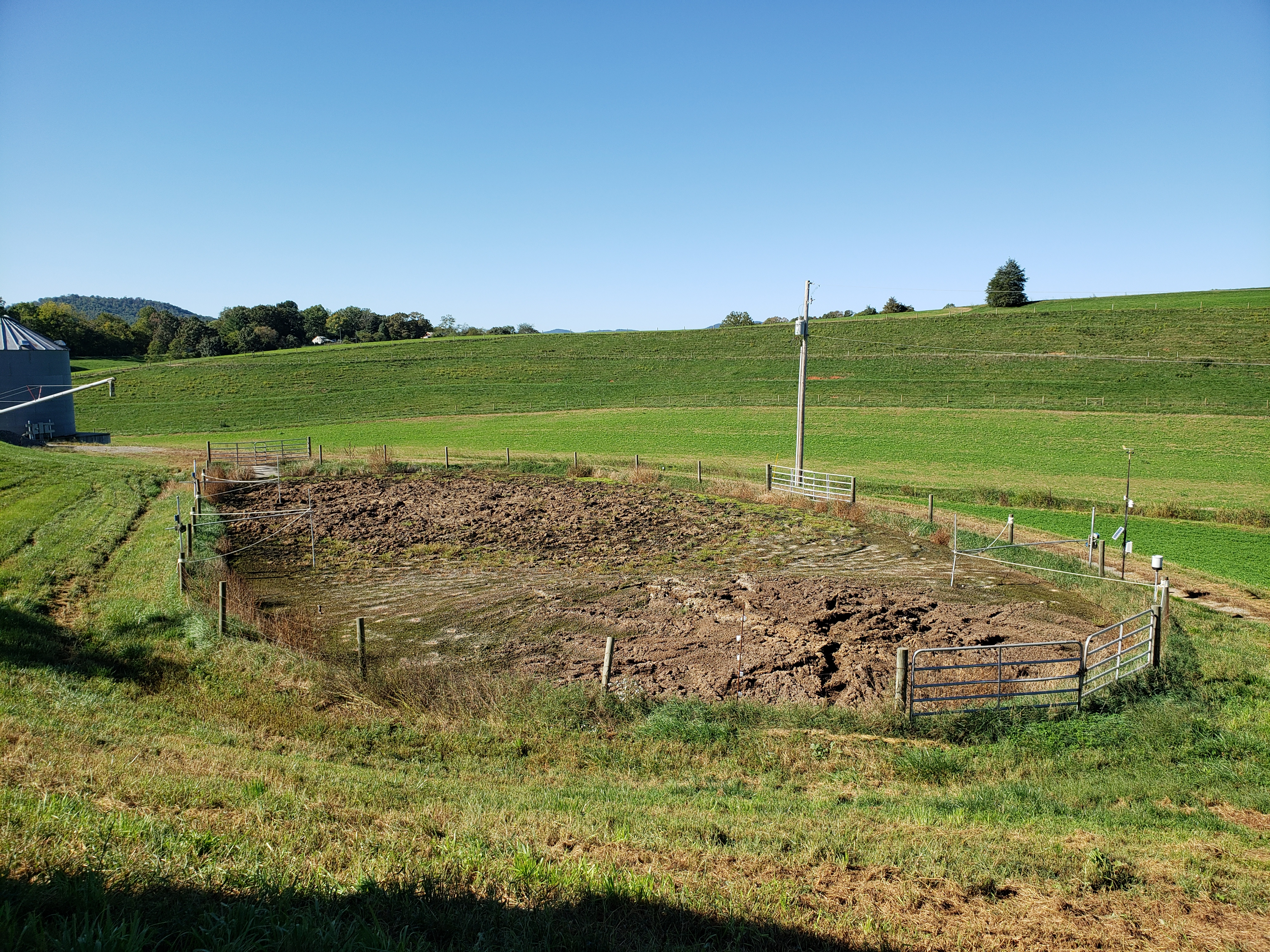

Supplement: S8 Fig — (TIFF) [file pone.0347665.s008.tiff]
